# LncRNA‐LOC101928316 contributes to gastric cancer progression through regulating PI3K‐Akt‐mTOR signaling pathway

**DOI:** 10.1002/cam4.2165

**Published:** 2019-06-17

**Authors:** Chengyun Li, Geyu Liang, Sheng Yang, Jing Sui, Wenjuan Wu, Siyi Xu, Yancheng Ye, Bo Shen, Xiaomei Zhang, Yan Zhang

**Affiliations:** ^1^ Department of Toxicology, School of Public Health Lanzhou University Lanzhou China; ^2^ Key Laboratory of Environmental Medicine Engineering, Ministry of Education, School of Public Health Southeast University Nanjing China; ^3^ Gansu Wuwei Tumor Hospital Wuwei China; ^4^ Department of Oncology Jiangsu Cancer Hospital Nanjing China

**Keywords:** gastric cancer, lncRNA, LOC101928316, molecular mechanism, progression

## Abstract

Long noncoding RNA (lncRNA) has played the important function in regulation of various biological processes and in diagnostic value has been widely appreciated. In the present study, we have found that LOC101928316 was significantly downregulated in gastric cancer (GC) tissues specimen, GC cell lines, and associated with the GC patients tumor, node, and metastasis (TNM) stage and degree of differentiation (*P* ＜ 0.05). LOC101928316 overexpression can significantly inhibit SGC‐7901 cell migration, invasion, and proliferation (*P*＜0.05). LOC101928316 molecular mechanism investigates suggested that LOC101928316 can regulate PI3K‐Akt‐mTOR signaling pathway and change the GC development progression in vivo and in vitro. In vivo experiment also revealed that LOC101928316‐Overexpression can inhibit the tumorigenicity of GC cells in tumor‐burdened experimental nude mice (*P *＜ 0.05). LOC101928316 may function as anti‐oncogene and also plays an important role in GC tumorigenesis. Collectively, our data provided the key role of LOC101928316 in the tumorigenesis of GC. In addition, the present study elucidates LOC101928316 potential regulatory network, which may help us to lead a better knowing of the pathogenesis of GC and probe the lncRNA as a novel biomarker to diagnosis and therapy for this malignant tumor.

## 1 INTRODUCTION

Gastric cancer (GC) is indeed a large threat to people health and with the incidence of this malignancy is in the second place in various cancers in China.^1^ Although the large advances in diagnostic and therapeutic applications, the prognosis for advanced GC is also poor, with a 5‐year survival rate of about 20%‐30%.^2^ To date, there were little GC molecular biomarkers have been investigated. Therefore, to improve the GC early stage diagnosis, efficiency and identification of the related biomarkers are urgent needed research directions.

Many evidences have revealed that long noncoding RNA (lncRNA) plays crucial roles in regulating the development and progression of GC.^3^ However, their specific functions and mechanisms remained to be further explored. Recently, plenty of studies have reported that the aberrantly expressed lncRNAs, including that of HOTAIR, H19, UCA1, MEG3, and MALAT1 significantly regulate GC cell cycle, cell proliferation, apoptosis, migration, invasion, metastasis, and tumorigenicity.^4^, ^5^ Meanwhile, due to the functional diversity of lncRNAs, identifying abnormally expressed lncRNAs in GC and deeply investigate the functions and mechanisms remains challenging.

Based on this status, our previous studies of the GC‐related lncRNAs differential expression profiles screening had used microarray analysis from advanced GC tissues and adjacent non‐tumor tissues, the GC‐related lncRNAs expression profiles showed that lncRNA LOC101928316 was significantly lower expression.^6^ However, the LOC101928316 is previously unknown about biological functions and mechanisms of target genes regulation. In this study, we investigated the LOC101928316 tissues expression levels in 90 GC tumor tissues and their paired adjacent noncancerous tissues then we further analyzed the potential relationships with clinical features.

Subsequently, according to the LOC101928316 expression levels of GC cells and cell location detection, we explored the LOC101928316 biological function of in vivo and in vitro through built vectors of overexpression and silencing lentivirus. In addition, our previous study also found the GC‐related mRNAs which were co‐expressed with the LOC101928316 and potential function as the regulator of the PI3K‐Akt‐mTOR signaling pathway.^6^ The PI3K‐AKT‐mTOR pathway is one of the key pathways in many human cancers such as esophagus cancer, lung cancer, breast cancer, liver cancer, and gastric cancer etc.^7^, ^8^, ^9^, ^10^ Meanwhile, there were more than 40 compounds that target key components of PI3K‐AKT‐mTOR pathway and have been investigated in clinical trials with a range of different cancers.^11^ Therefore, in present study, we attempted to characterize the biological function of LOC101928316 on the PI3K‐Akt‐mTOR pathway in GC cells, and evaluated whether LOC101928316 exerted antitumor actions in GC. Our study sheds a light upon the function of LOC101928316 and its role in GC diagnosis and provides a new evidence for further clinical biomarker investigation.

## 2 MATERIALS AND METHODS

### 2.1 Patients and tissue samples

In this study, there were 90 GC tumor and paired non‐tumor tissue samples obtained from GC patients whom diagnosed with the pathology after surgery in Wuwei Tumor Hospital of Gansu, between 2015 and 2017. In addition, all included GC patients underwent radical operation and had no radiotherapy or chemotherapy history before surgery. These GC patients were diagnosed based on the Cancer Staging Manual. Meanwhile, GC patients' clinicopathological variables were recorded and the information including age, gender, tumor size, tumor grade, tumor, node and metastasis (TNM) stage, and lymph node invasion status. Tissues samples were immediately immersed in RNAlater^®^ (Ambion) and stored at −80°C. This study was approved by the Ethics Committee of Wuwei Tumor Hospital of Gansu with informed consent from patients.

### 2.2 Cell lines, culture, and irradiation

The human GC cell lines including MKN‐28, BGC‐823, SGC‐7901, MGC‐803, and MKN‐45, and the human normal gastric epithelium cell line (GES‐1), all were obtained from the Shanghai Cell Bank of the Chinese Academy of Science. All of the above cell lines were cultured in DMEM (GE Health Care HyClone™) medium and supplemented with 10% fetal bovine serum (FBS), 100 units/mL of penicillin, and 100 mg/mL of streptomycin. Cells were cultured at 37°C in humidified air with 5% CO_2_.

### 2.3 RNA isolation and qRT‐PCR analyses

Total RNAs were extracted from tissues and cells using TRIzol^®^ reagent (Invitrogen) according to the manufacture's instruction. RNAs reverse‐transcribed to cDNA using Reverse Transcription Kit (Promega) and through real‐time PCR to detect the expression levels of LOC101928316 using the Step One Plus^TM^ PCR System (Applied Biosystems), and GoTaq^®^ qPCR Master Mix of Power SYBR^®^ Green (Promega) was used for qRT‐PCR detection. The Loc101928316 sense primer was 5′‐ AACAACGGGGACATTAGG‐3′ and the reverse primer was 5′‐ AACTGGAAACATCACATAGCA‐3′; meanwhile, results were normalized by GAPDH, the GAPDH sense primer was 5′‐GGGAGCCAAAAGGGTCATCA‐3′ and the reverse primer was 5′‐ TGATGGCATGGACTGTGGTC‐3′. After 40 cycles, data were analyzed by 2^−ΔΔ^ *^C^* ^t^ method.

### 2.4 Association analyses between LOC101928316 expression levels and clinical features

We analyze the association between LOC101928316 expression levels and GC patients' clinical features, and the indicators including the tumor sizes, TNM stage, tumor grade, and lymphatic metastases invasion status.

### 2.5 In situ hybridization

The RNA in situ hybridization probe (LOC101928316 gene location, 3734 ~ 4270) was synthesized from Exon Biotech, Guangzhou, China. We used plasmid containing T3 and T7 promoters to reverse transcriptase, and DIG‐dUTP was incorporated to probe the sequence. Experiment was performed according to the probe manufacturer's protocol. Finally, all the resulting images were observed by the FSX100 (Olympus) microscope system.

### 2.6 Plasmid transfection

The LOC101928316 vectors were constructed at Abmgood Inc Zhenjiang, China. In this study, LOC101928316 upregulated and downregulated cells transfection through the Lenti‐CMV‐GFP‐2A‐Puro‐LOC101928316 and the piLenti‐LOC101928316‐siRNA‐GFP lentivirus vector. Cells were cultured in the 6‐well plates and transfected with Lenti‐CMV‐GFP‐2A‐Puro‐LOC101928316, piLenti‐LOC101928316‐siRNA‐GFP, according to the reagents manufacturer's protocols. Subsequently, stably transfected cell lines were screened by puromycin. Finally, the stable transfection cell lines expression level of LOC101928316 was detected by qRT‐PCR.

### 2.7 Cell proliferation and wound healing assays

To investigate the effect of cell proliferation by lentivirus transfected, the 3‐(4,5‐dimethylthiazol‐2‐yl)‐2,5‐diphenyltetrazolium bromide (MTT) assay was used to detect the cell proliferation Kit (Sigma‐Aldrich) according to the manufacturer's protocol. Cells were seeded into each well of a 96‐well plate and incubated for 12, 24, 48, and 72 hours. Following addition of 200 μL which containing 0.5 mg/mL MTT to the plate, and incubated for 4 hours at 37℃ in thermotank. The next step discarded the medium and added 100 µL DMSO into each well, and incubated for 10 minutes in the dark. Finally, the absorbance values were detected at 490 nm by the microplate reader (Bio Tek Instruments Inc).

Cell migration was observed through the wound healing assay. First, cells were seeded into 6‐well plates and cultured normally, when cells individual wells reached 90% confluency, and the scratch was made in the plate using a sterile 200 μL pipette tip. Subsequently, detached cells were washed with PBS, and the cells were incubated with 5% CO_2_ at 37°C. Finally, microscope photographs were taken at different time node, photographed using a digital camera system (FSX100 Biological Image system, Olympus). Three independent experiments were performed.

### 2.8 Invasion assay

Transwell^®^ Matrigel^®^ system was used to observe the cell invasion. For the invasion assay, 1 × 10^5^ cells were seeded into the 24‐well Transwell^®^ plates upper chamber (Corning), which insert coated with Matrigel (BD Biosciences). After incubating for 24 hours, cells on the bottom side of the upper chambers' membrane were fixed by 4% polyoxymethylene for 15 minutes, then stained by crystal violet dye for 10 minutes, and finally visualized using the FSX100 microscope (Olympus).

### 2.9 Flow cytometry assay

Apoptosis was evaluated using an Annexin V‐APC 7AAD apoptosis kit (MultiSciences Biotech). Flow cytometer (BD Biosciences) was used to quantify apoptosis detection and data analysis. Q1 represent dead cells, Q2 represent early apoptotic cells, Q3 represent viable cells, and Q4 represent late apoptotic cells.

### 2.10 Western blotting assay and antibodies

Western blotting experiment was used to evaluate the expression levels of proteins in different cell lines. SDS‐PAGE was used to isolate proteins, and transferred to nitrocellulose membrane (PVDF), subsequently. The membrane was blocked for 2 hours in 5% skimmed milk, and then primary antibodies were incubated at 4°C using 5% bovine serum albumen (BSA) for 12 hours, followed using corresponding secondary antibodies incubated for 1 hour. The protein signals were detected by ECL chemiluminescence system (Thermo Fisher Scientific). All above antibodies were purchased from Cell Signaling Technology, and the experiments were repeated three times.

### 2.11 Animal experiments

BALB/c nude mice (4‐week‐old, male, pathogen‐free conditions) were inoculated subcutaneously with 5 × 10^7^ cells/mouse. Tumors growth was examined every 5 days, and the volumes were evaluated with the formula: V = (L × W^2^)/2, (L, longitudinal diameter; W, latitudinal diameter). At the end of the experiment (21 days later), all the nude mice were euthanized and the tumors were isolated. Finally, the expression levels of LOC101928316 and key protein were detected from the tumor specimens by qRT‐PCR and immunohistochemical analyses. Animal maintenance and experimental procedures were approved by the Committee on the Ethics of Animal Experiments of Lanzhou University.

### 2.12 Statistical analysis

Statistical analyses were performed using SPSS 21.0 software (IBM Corp.) and Excel software. The results were expressed as mean ± SD. Statistical significance was determined using the Student's *t* test or the Chi‐squared test as appropriate.

## 3 RESULTS

### 3.1 The expression of LOC101928316 was downregulated and correlated with the degree of differentiation and TNM stage

**Figure 1:**
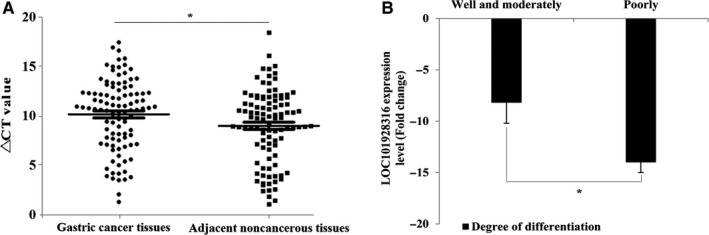
LOC101928316 expression levels of gastric cancer tissues samples. A, Comparison between 90 cancer tissues and paired adjacent noncancerous tissues, Δ*C*_t_ = (Ct RNAs – Ct GAPDH) ^*^ *P *＜ 0.05; B, LOC101928316 downregulation fold change between degrees of differentiation well and poorly, ^*^ *P* ＜ 0.05)

The associations between the expression level of the LOC101928316 in 90‐paired GC samples and the clinicopathological characteristics of these recruited GC patients were analyzed. As shown in Figure 1A, in GC tissues, LOC101928316 expression level was lower than the average of adjacent non‐tumor tissue samples. The results suggested that decreased LOC101928316 expression was correlated with GC degree of differentiation and TNM stage, and with no correlation between LOC101928316 and other GC clinical pathological features in this study (Table 1). Finally, we analyzed the changes of LOC101928316 expression in the degrees of differentiation well and poorly. The results revealed that the LOC101928316 expression levels showed a significant gradual decrease from the degrees of differentiation well to poorly of GC (Figure 1B).

**Table 1:** The correlations between LOC101928316 and gastric cancer clinical features

Variable	Cases, n (%)	LOC101928316		Chi‐squared test χ^2^‐value	*P*‐value
		Low‐LOC101928316 group, no. of cases	High‐LOC101928316 group, no. of cases		
Gender					
Male	63 (70)	49	14	0.521	0.470
Female	27 (30)	22	9		
Age, (y)					
≤50	20 (22)	16	4	0.417	0.518
>50	70 (78)	51	19		
Tumor size, cm					
≤5	50 (56)	38	12	0.143	0.705
>5	40 (44)	29	11		
Degree of differentiation					
Well and moderately	29 (32)	17	12	5.631	0.018*
Poorly	61 (68)	50	11		
TNM stage					
I/II	48 (53)	31	17	5.257	0.022*
III/Ⅳ	42 (47)	36	6		
Lymph‐node status					
No metastasis	42 (47)	28	14	2.504	0.114
Metastasis	48 (53)	39	9		

Note

Abbreviation: lncRNA, long noncoding RNA; TNM, tumor node metastasis.

* *P* < 0.05.

### 3.2 LOC101928316 exist in the cytoplasm and was downregulated in gastric cancer cells

**Figure 2:**
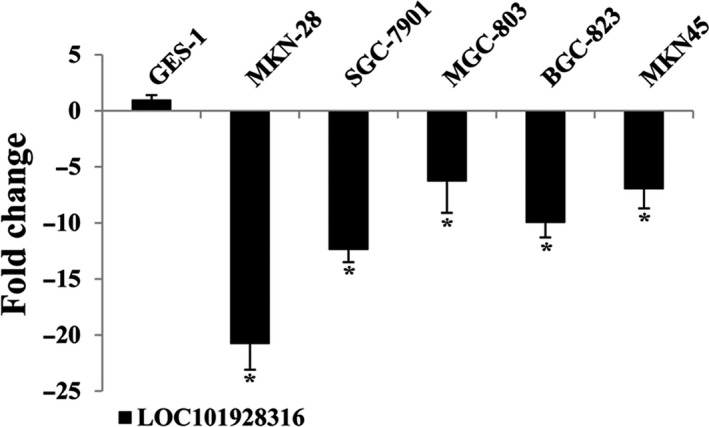
LOC101928316 relative expression levels between gastric cancer cell lines and normal gastric mucosa cell line, respectively. (*P ＜ 0.05; **P ＜ 0.01)

**Figure 3:**
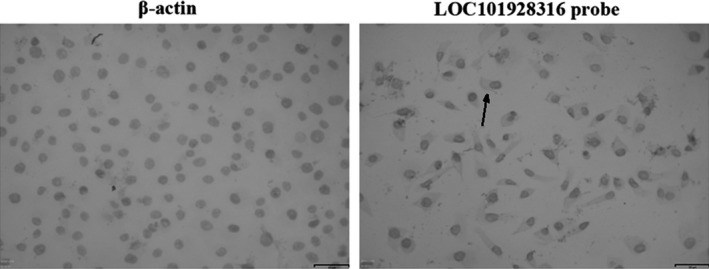
RNA in situ hybridization of LOC101928316 in SGC‐7901 cells

Five GC cell lines (MKN‐28, SGC‐7901, MGC‐803, BGC‐823, MKN‐45) and the human normal gastric epithelium cell line (GES‐1) were selected and detected their expressions of LOC101928316 by qRT‐PCR. Results showed that LOC101928316 expression levels were significantly downregulated in GC cell lines which compared with the GES‐1 cell line (Figure 2). Integrated analyses for above results suggested that LOC101928316 expression level was frequently lower in GC tissues and GC cell lines. Meanwhile, RNA in situ hybridization results showed that LOC101928316 can be observed in the cytoplasm of SGC‐7901 (Figure 3), and suggested that downregulation of LOC101928316 may regulate the gene functions in transcriptional regulation.

### 3.3 Lentivirus‐mediated infection altered the in vitro expression of LOC101928316

**Figure 4:**
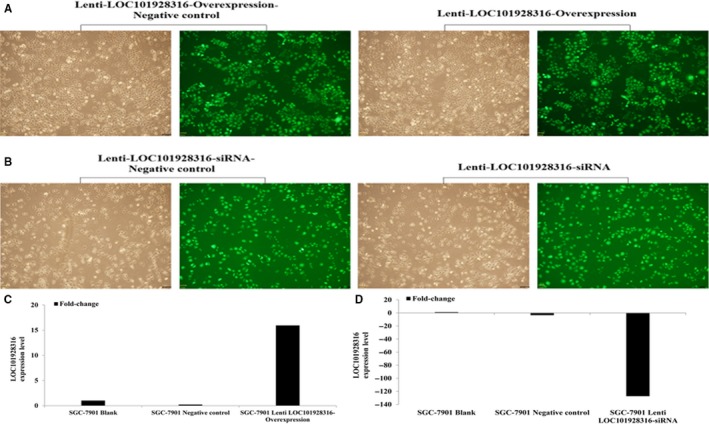
Lentivirus stabilization transfected SGC‐7901 cells efficiency (A‐B), qRT‐PCR detected ((C) SGC‐7901 Lenti‐LOC101928316‐Overexpression; (D) SGC‐7901 Lenti‐LOC101928316‐ siRNA)

To investigate the biological role of LOC101928316 in GC, lentivirus vectors were constructed and infected the human SGC‐7901 cell line, according to the reagents manufacturer's protocol. Subsequently, puromycin was used to screen the lentivirus stably transfected GC cell lines (Figure 4A and B). LOC101928316 expression levels detection results showed that the lentivirus‐LOC101928316‐Overexpression transfected SGC‐7901 cell line, the LOC101928316 expression was upregulated 15.96‐fold; and the lentivirus‐LOC101928316‐siRNA transfected SGC‐7901, the LOC101928316 was downregulated 127.41‐fold, which compared with the blank and negative control (Figure 4C and D).

### 3.4 The effect of LOC101928316 on gastric cancer cell proliferation and apoptosis in vitro

**Figure 5:**
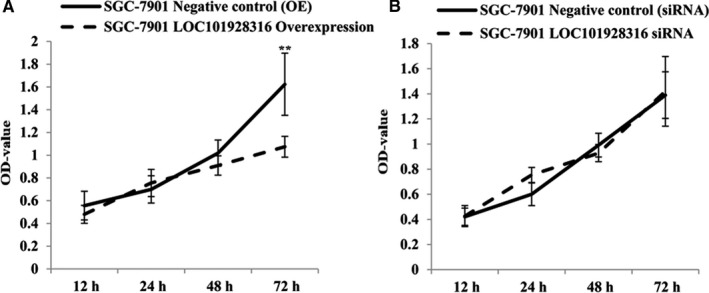
Lentivirus stable transfected SGC‐7901 cells, the proliferation was detected which incubated for 12, 24, 48, and 72 h, (A) Lentivirus‐LOC101928316‐Overexpression; (B) Lentivirus‐LOC101928316‐siRNA). MTT assay results values present mean ± SD by three independent experiments (^*^ *P* ＜ 0.05; Lentivirus LOC101928316 overexpression and siRNA stable transfected SGC‐7901 cells vs Negative control, NC)

**Figure 6:**
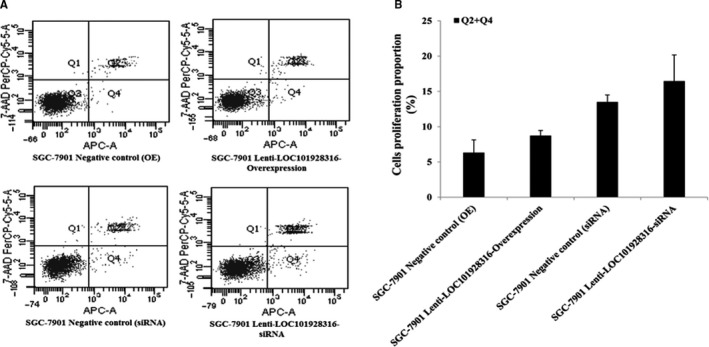
Apoptosis detection in the lentivirus LOC101928316 overexpression and siRNA stable transfected SGC‐7901 cell via. A, Apoptosis assays detection results figures; B, Stable transfected SGC‐7901 cell vs corresponding negative control. Results value present mean ± SD; (n ≥ 3) of the samples

The MTT assay and flow cytometry assay were used to detect whether LOC101928316 could affect GC cell proliferation and apoptosis. MTT detection results revealed that SGC‐7901 growth was decreased significantly in the lentivirus‐LOC101928316‐Overexpression group after 48 hours, which compared with the negative control cell line (*P* < 0.01; Figure 5A). Lentivirus‐LOC101928316‐siRNA cells, SGC‐7901 cell growth no significant difference with negative control group (*P* ＞ 0.05; Figure 5B). Apoptosis of the stably transfected SGC‐7901 cells was determined by flow cytometry (Figure 6), and the results showed that SGC‐7901 cells apoptosis was no significant different compared with the negative control group (*P* ＞ 0.05; Figure 6B).

### 3.5 LOC101928316‐Overexpression decreased the migration and invasion of gastric cells

**Figure 7:**
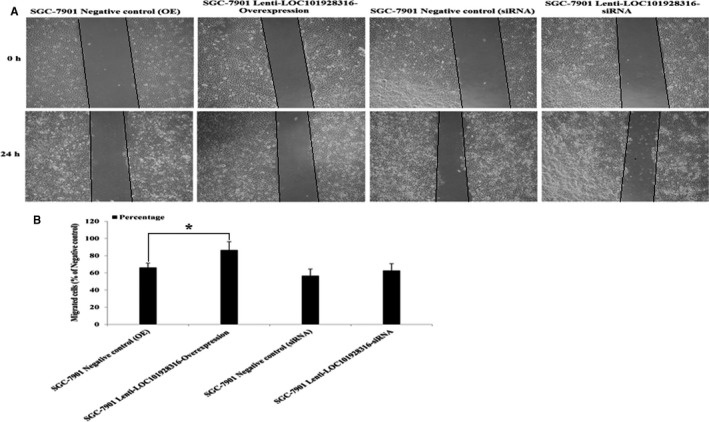
Effects on GC cell migration ability of LOC101928316: A, Representative images of SGC‐7901 cell lentivirus stable transfected at 0 and 24 h of wound healing assay; B, Image pro plus was used to analyze the results of wound scratch images (*P ＜ 0.05)

**Figure 8:**
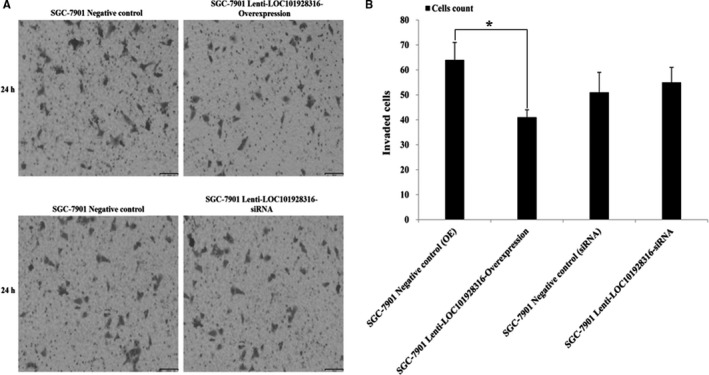
Effects on GC cell invasion of LOC101928316: A, Representative images of crystal violet stained for lentivirus stable transfected of SGC‐7901 cell by transwell invasion assays; B, The results value present mean ± SD; (n ≥ 6) (^*^ *P* ＜ 0.05)

The result of LOC101928316 was downregulated and correlated with the degree of differentiation and TNM stage in 90 GC patients. We also investigate whether the LOC101928316 has function as important role in migration and invasion of GC cells. Migration and invasion capability were detected using wound healing assay and Transwell^®^ invasion assay. Figure 7A showed that SGC‐7901 stably infected by lenti‐LOC101928316‐Overexpression, the migration decreased significantly which compared with negative control (*P* < 0.05). In addition, Transwell^®^ assay also revealed similar results when stable infection by the lenti‐LOC101928316‐Overexpression. Lenti‐LOC101928316‐Overexpression stable infected lowered the SGC‐7901 invading cells number, which compared with negative group (Figure 8B). Above results suggested that LOC101928316 overexpression can inhibit GC cell migration and invasion.

### 3.6 LOC101928316 regulated PI3K‐Akt‐mTOR signaling pathway and their downstream mediators

**Figure 9:**
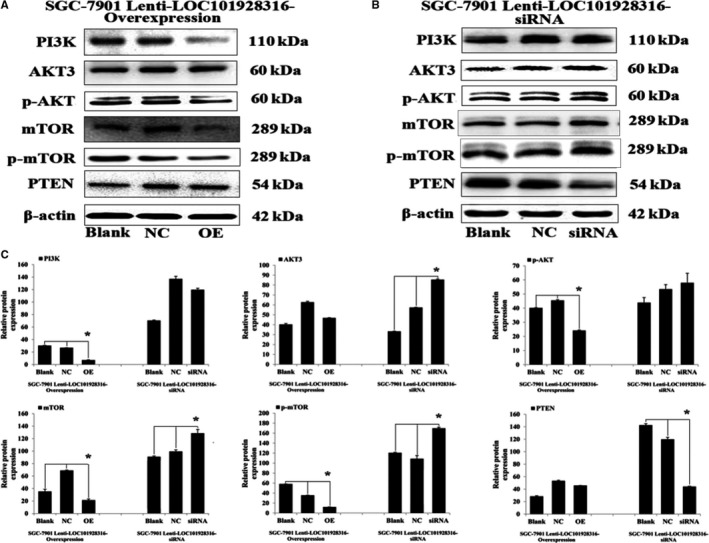
Western blotting results of protein expression from lenti‐LOC101928316 overexpression (A) and siRNA (B) stable transfected SGC‐7901 cells. C, Image pro plus was used to analyze the relative optical density results of western blotting. ^*^ *P* < 0.05

**Figure 10:**
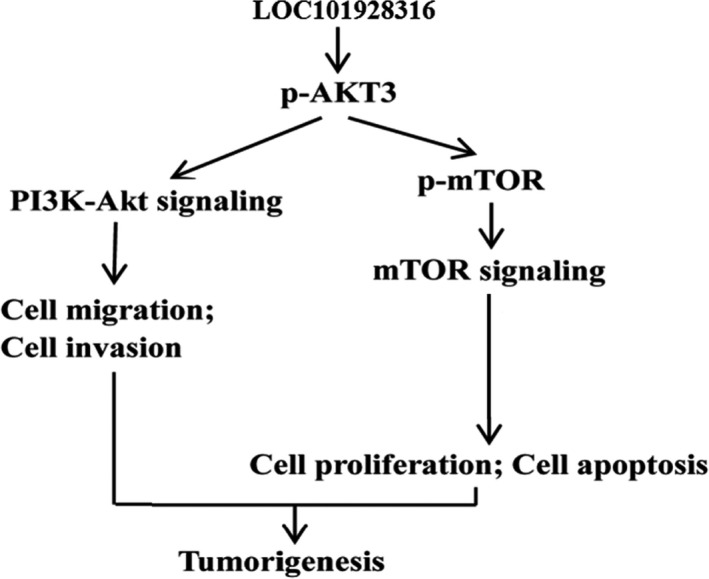
LOC101928316 potential regulation mechanism speculates in the tumorigenesis progression of gastric cancer

PI3K‐AKT‐mTOR signaling pathway which is dysregulated in many cancer types and affects cell proliferation, apoptosis, energy metabolism etc.^12^ Our previous study^6^ has found that some of the LOC101928316 co‐expressed mRNAs which were involved in PI3K‐Akt‐mTOR signal pathway, so we explored whether the LOC101928316 can regulation of the PI3K‐Akt‐mTOR pathway. Western blotting results revealed that lenti‐LOC101928316‐Overexpression stable infected can remarkably inhibit the expression level of PI3K, p‐AKT, mTOR, and p‐mTOR in SGC‐7901 cells, which compared with blank and negative control groups, respectively (*P* ＜ 0.05; Figure 9A and C); Meanwhile, lenti‐LOC101928316‐siRNA stable infected can remarkably increase the expression level of AKT3, mTOR, p‐mTOR, and inhibited the expression level of PTEN in SGC‐7901 cells, respectively (*P* ＜ 0.05; Figure 9B and C). Together, the above results revealed that LOC101928316 can affect the I3K‐Akt‐mTOR signaling pathway and change the malignant progression of GC cells (Figure 10).

### 3.7 LOC101928316‐Overexpression inhibited gastric cancer cells tumorigenesis in vivo

**Figure 11:**
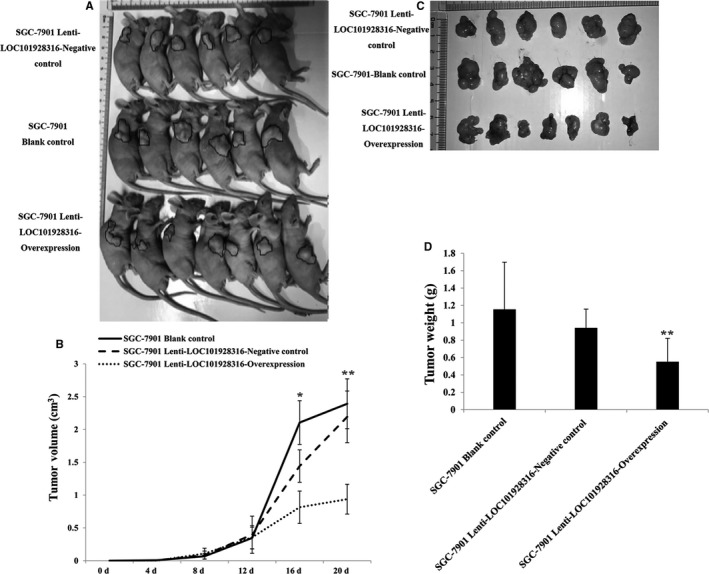
A, Effects of LOC101928316 on tumor growth in vivo; B, Volume of the subcutaneous tumors at 4 to 20 d. C‐D, Images of tumor tissues, SGC‐7901 lenti‐LOC101928316‐Overexpression group the tumors were smaller than blank and negative control groups

**Figure 12:**
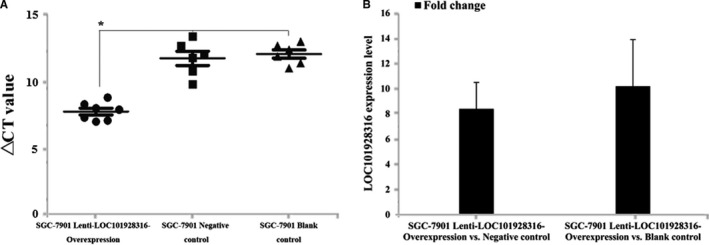
Tumor tissues LOC101928316 expressions in vivo of nude mice using qRT‐PCR; A, △CT value comparison between SGC‐7901 lenti‐LOC101928316‐Overexpression, negative and blank control groups; B, LOC101928316 expressions fold change of SGC‐7901 lenti‐LOC101928316‐Overexpression vs Negative control and SGC‐7901 lenti‐LOC101928316‐Overexpression vs Blank

**Figure 13:**
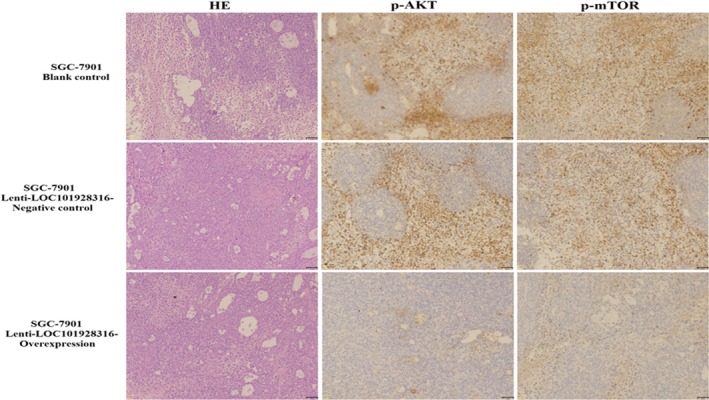
Immunohistochemical analysis of the proteins expression levels of p‐AKT and p‐mTOR in vivo of nude mice tumor tissues

To investigate whether the expression of LOC101928316 can affect the GC progression, SGC‐7901 cells blank, lenti‐LOC101928316‐Overexpression, and empty vector stably transfected SGC‐7901 cells were injected into nude mice's subcutaneous, respectively. Three weeks later, the average size of tumors in the LOC101928316 overexpression group was significantly smaller than negative control group and blank group (Figure 11A and B). Average tumor weight detected at the end of the experiment was markedly reduced in the lenti‐LOC101928316‐Overexpression group (0.55 ± 0.27 g) compared with the blank group (0.94 ± 0.22 g) and the empty vector group (1.16 ± 0.54 g; *P *＜ 0.05; Figure 11C and D). Tumor‐bearing nude mice assay tumor tissues was isolated, and the LOC101928316 expression was detected by qRT‐PCR. We found that LOC101928316 expression levels in tissues formed by LOC101928316 overexpression group were higher than negative and blank groups (Figure 12A and B). The p‐AKT3 and p‐mTOR proteins expression levels in isolated tumor tissues also were analyzed by immunohistochemical. Results suggested that lenti‐LOC101928316‐Overexpression stable transfected SGC‐7901 cells can significantly inhibit the p‐AKT and p‐mTOR proteins expression levels which compared with SGC‐7901 blank and negative groups (Figure 13). Comprehensively analyzing the above results, we found that LOC101928316 overexpression can inhibit the GC tumorigenesis in vivo.

## 4 DISCUSSION

Despite the advances in therapeutic approaches, including surgical methods, chemotherapy, and radiotherapy for GC, the prognosis for patients who diagnosed in an advanced stage also is poor.^13^ Thus, molecular mechanisms underlying GC progression are urgently needed for deep investigation to provide better biomarkers of diagnosis and therapeutic treatments for GC. Recent studies have suggested that lncRNAs have played crucial roles in cancer progression.^14^ LncRNAs can regulate metabolism, cell differentiation, and individual development progression of life on multiple levels and related with human serious diseases.^15^, ^16^, ^17^ LOC101928316 genome which is located on the chromosome 11p14.1 have reported to be significantly differentially lower expressed in GC tumor tissues in our previous study and this result suggest that the LOC101928316 gene may play important roles in GC tumorigenesis, but the mechanism of functions is still undiscovered.^6^

In this study, the LOC101928316 expression levels of 90 GC patients' tumor tissues samples and their paired non‐cancerous tissues were detected by qRT‐PCR, and then correlated the GC patients' LOC101928316 expression results with their clinical pathological features. Integrated analysis the correlation, our results revealed that the LOC101928316 was downregulated in GC tumor tissues and may function as an anti‐oncogene in GC progression. Subsequently, we analyzed the LOC101928316 expression and GC patients' clinical pathological features, and found that LOC101928316 was closely related with the GC patients' degree of differentiation and TNM stage. Therefore, LOC101928316 may be a potential anti‐oncogene and plays the important role in GC tumorigenesis.

To explore the possibility in biological functions regulation of LOC101928316 in GC, the LOC101928316 low expression SGC‐7901 cell line was selected. In the present study, we have observed that LOC101928316 gene can be found in SGC‐7901 cell cytoplasm, suggested that downregulation of LOC101928316 may function as key role in transcriptional and posttranscriptional regulation. Subsequently, we investigate whether LOC101928316 can affect the major functions of SGC‐7901 cells, through lentivirus LOC101928316 overexpression and siRNA stably transfected methods. MTT assay results revealed that SGC‐7901 cell growth was significantly slower in lenti‐LOC101928316‐Overexpression group, which compared with negative control. The results suggested that LOC101928316 can regulate GC cell proliferation at different expression levels; however, the relationship between LOC101928316 and GC development still remains unknown.

Our study also found that the LOC101928316 expression level was significant gradual decrease from the degrees of differentiation well to poorly of GC, revealed that low expression of LOC101928316 may inhibit GC progression and development. Therefore, we investigate whether LOC101928316 can effect the progression and development of GC cells in vitro. Lenti‐LOC101928316‐Overexpression stable infection SGC‐7901 cell, the Wound healing assay and Transwell^®^ invasion assay revealed that infected SGC‐7901 cell migration and invasion both were decreased; meanwhile lenti‐LOC101928316‐Overexpression stable infection can lower the number of SGC‐7901 invading cells. Results showed that SGC‐7901 cell migration and invasion were inhibited through LOC101928316 overexpression, suggesting that we can use LOC101928316 as a gene‐specific target for GC treatment.

To explore the regulation of molecular mechanism of LOC101928316 for GC cells proliferation, invasion, and migration, we analyzed the expression of closely associated proteins expression levels for the above indicators. In the previous study, we have found that some of the GC‐related mRNAs such as AKT3 which were co‐expressed with LOC101928316 and regulated the PI3K‐Akt‐mTOR signal transduction. The PI3K‐AKT‐mTOR pathway is an intracellular signaling pathway important in regulating the cell cycle, apoptosis, growth, and other cell biology procession.^18^, ^19^ We confirmed that LOC101928316 overexpression stable infected can remarkably inhibit the expression level of PI3K, p‐AKT, mTOR, and p‐mTOR in SGC‐7901 cells in this study. In addition, LOC101928316‐siRNA can increase the expression level of AKT3, mTOR, p‐mTOR, and inhibit the expression level of PTEN in SGC‐7901 cells. Subsequently, we use an immunohistochemical assay to determine the protein expression of p‐AKT3 and p‐mTOR from nude mice tumor tissues. Tumor‐bearing nude mice assay results showed that LOC101928316 overexpression can significantly inhibit the p‐AKT3 and p‐mTOR protein expression levels in bearing nude tumor tissues. In the present study, we can find the LOC101928316 overexpression inhibited the PI3K‐Akt‐mTOR pathway and LOC101928316 silencing activated this signaling pathway. PI3K‐AKT‐mTOR signaling pathway has function as essential role in the carcinogenesis progression regulation of common cancers.^20^, ^21^, ^22^ In this study, the overexpression and siRNA of LOC101928316 can affect the p‐AKT3 and p‐mTOR expression levels, and these key proteins can regulate the carcinogenesis biological processes. Current study also have reported that several lncRNAs can affect PI3K‐Akt‐mTOR signaling pathway key proteins expression such as breast cancer,^23^, ^24^ cervical cancer,^25^, ^26^ and non‐small cell lung cancer,^27^ but similar studies about LOC101928316 for GC have not been reported.

Finally, the tumorigenesis progression and expression levels of LOC101928316, p‐AKT3, and p‐mTOR were observed by lenti‐LOC101928316‐Overexpression in vivo. We found that tumors formed in LOC101928316‐overexpressed male nude mice group were significantly smaller than blank and negative control groups. Functional assays were shown the consistent results in the tumorigenesis inhibition of LOC101928316 overexpressed. However, similar studies of the LOC101928316 have not been reported fully at present. Our results suggested that overexpression of LOC101928316 can inhibit GC tumor growth progression in vivo.

In conclusion, this study has identified the potential functions of LOC101928316 in GC tumorigenesis. We found that LOC101928316 was significantly downregulated in GC tissues and GC cell lines. Correlation analysis showed that downregulated LOC101928316 correlated with the degree of differentiation and TNM stage. Moreover, functional assay suggested that LOC101928316 overexpression significantly inhibited GC SGC‐7901 cell proliferation. We also found that LOC101928316 might play a potential role in GC cells migration and invasion both in vitro and in vivo. The mechanism of LOC101928316 regulation in PI3K‐Akt‐mTOR pathway suggested that LOC101928316 alters GC progression in vitro and in vivo. Importantly, our study systematically investigates the relationship and potential regulation mechanism between LOC101928316 and GC. Our results suggested that GC tissues downregulated lncRNA LOC101928316 may be potential biomarker for diagnosis and prognosis of GC, which is an interesting indicator for further research.

## ETHICS APPROVAL AND CONSENT TO PARTICIPATE

The present study was approved by the Ethics Committee of the Gansu Wuwei Tumor Hospital. All patients provided written informed consent to participate in the present study.

## CONFLICT OF INTEREST

The authors declare that they have no competing interests.

## AUTHORS' CONTRIBUTIONS

CYL and GYL conceived and designed the study. CYL and SY performed the experiments. JS, WJW, and SYX analyzed and interpreted the results. YCY, SB, XMZ, and YZ performed the gastric cancer patients' tissue sample collection and quality control. CYL and GYL performed analysis and quality control, and were major contributors in writing the manuscript. All authors read and approved the final manuscript.

## CONSENT FOR PUBLICATION

All participants confirmed that the data can be published.

## DATA AVAILABILITY

The authors declare that all relevant raw data will be made freely available to any researchers who wish to use them for non‐commercial purposes, whilst preserving any necessary confidentiality and anonymity. All data generated or analyzed during this study are included in this published article.
